# Supplementary material for: Cross-species oncogenic signatures of breast cancer in canine mammary tumors
Source: Nat Commun. 2020 Jul 17;11:3616. doi: 10.1038/s41467-020-17458-0 (PMC7367841; doi:10.1038/s41467-020-17458-0)
Supplement: Supplementary file 1 — Supplementary Information [file 41467_2020_17458_MOESM1_ESM.pdf]

Supplementary Information for

Cross-species Oncogenic Signatures of Breast Cancer in Canine Mammary Tumors

Tae-Min Kim[1], In Seok Yang[2], Byung-Joon Seung[3], Sejoon Lee[4], Dohyun Kim[5], Yoo-Jin Ha[2], Mi-Kyoung Seo[2], Ka-Kyung Kim[2], Hyun Seok Kim[6], Jae-Ho Cheong[2], Jung-Hyang Sur[3], Hojung Nam[5], Sangwoo Kim[2,*]

[1] Department of Medical Informatics and Cancer Research Institute, College of Medicine, The Catholic University of Korea, Seoul 06591, South Korea

[2] Department of Biomedical Systems Informatics and Brain Korea 21 PLUS in Medical Science, Yonsei University College of Medicine, Seoul 03722, South Korea

[3] Department of Veterinary Pathology, Small Animal Tumor Diagnostic Center, College of Veterinary Medicine, Konkuk University, Seoul 05029, South Korea.

[4] Department of Pathology and Translational Medicine, Seoul National University Bundang Hospital, Seongnam 13620, South Korea

[5] School of Electrical Engineering and Computer Science, Gwangju Institute of Science and Technology (GIST), Gwangju 61005, South Korea

[6] Severance Biomedical Science Institute, Yonsei University College of Medicine, Seoul 03722, South Korea

* To whom correspondence should be addressed: swkim@yuhs.ac

Supplementary Figures

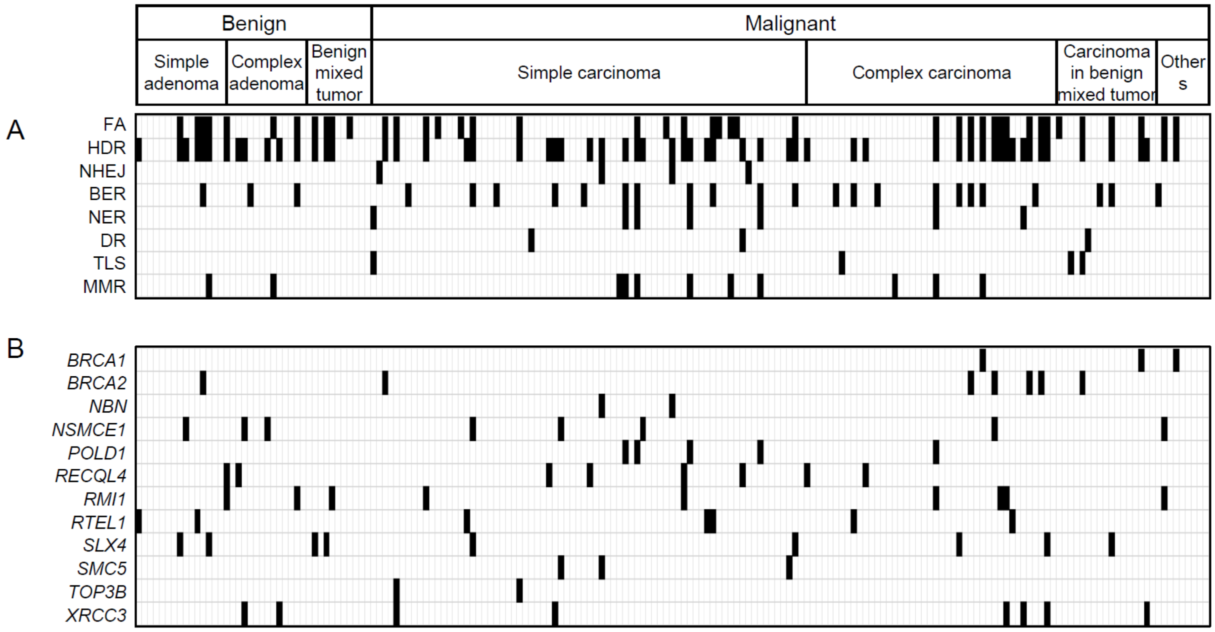

Supplementary Figure 1. Germline predisposing variants in CMT genomes. (A) For 8 DNA damage and repair pathway, cases harboring germline variants are marked. The abbreviation of pathways are Homology dependent recombination (HDR), Fanconi Anemia (FA), Direct Repair (DR), Non-homologous End Joining (NHEJ), Mismatch Repair (MMR), Base Excision Repair (BER), Translesion Synthesis (TLS), Nucleotide Excision Repair (NER). (B) For HR pathway, 12 genes with germline variants are demonstrated.

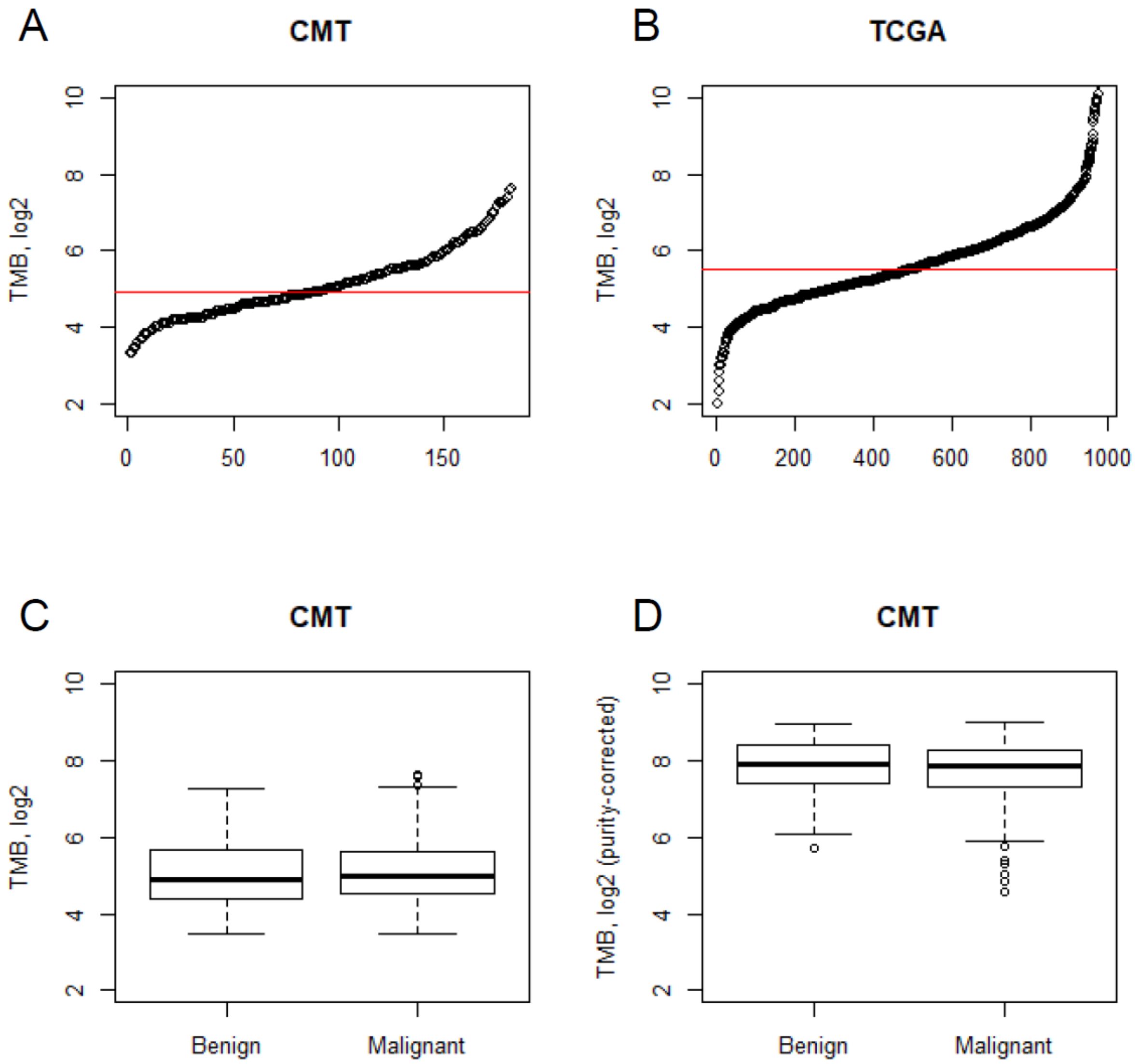

Supplementary Figure 2. Mutation burden of CMT and human breast cancers. (A) TMB (log_2 scaled) of 183 CMT cases are shown with a line indicating the median value. (B) Similarly shown for TMB of human breast cancers (TCGA consortium) are shown. (C) TMB values are shown for 40 benign and 143 malignant CMT, respectively. (D) Also shown for the comparison of purity-adjusted TMB between 40 benign and 143 malignant CMTs.

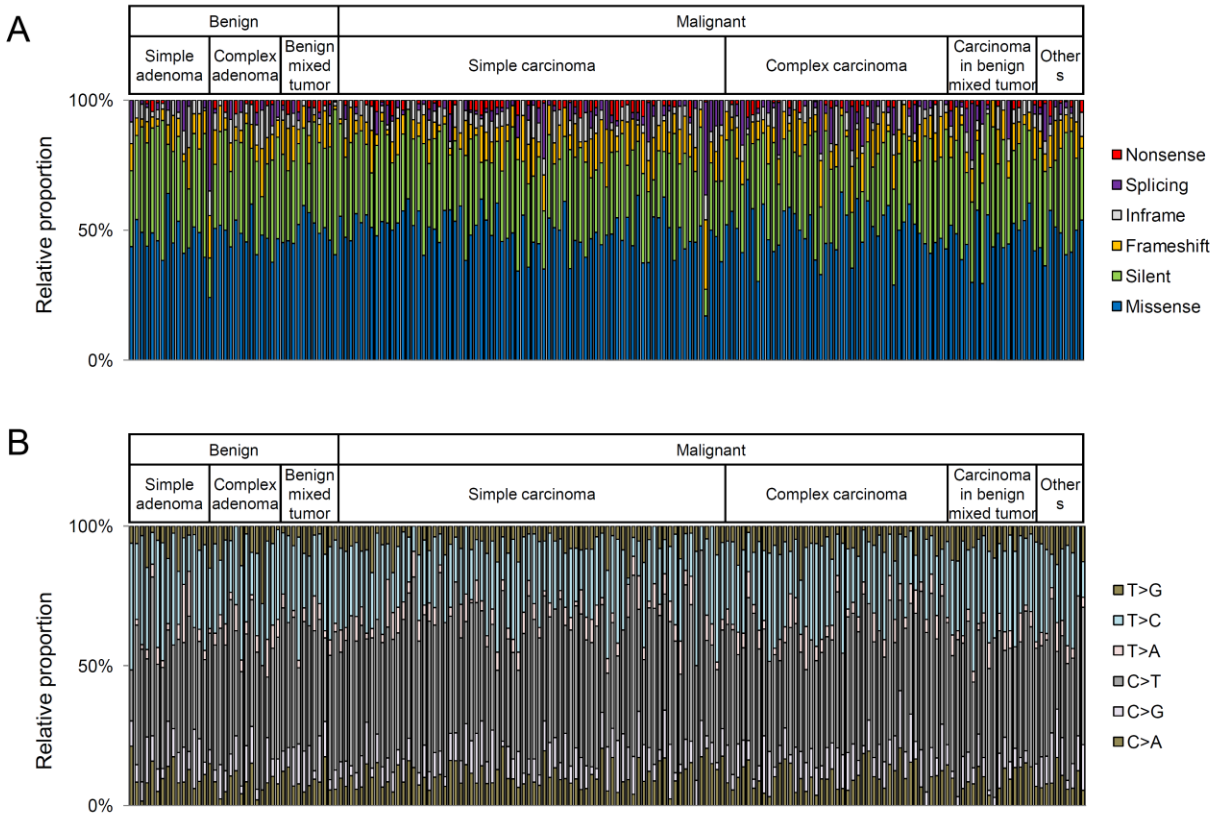

Supplementary Figure 3. Functional consequences and mutation spectra of CMT. (A) The impact of somatic mutations on coding amino acids is presented as relative proportions (%). The order of the 183 CMTs is the same as that in main Figure 1. (B) The six mutation spectra for individual cases are shown.

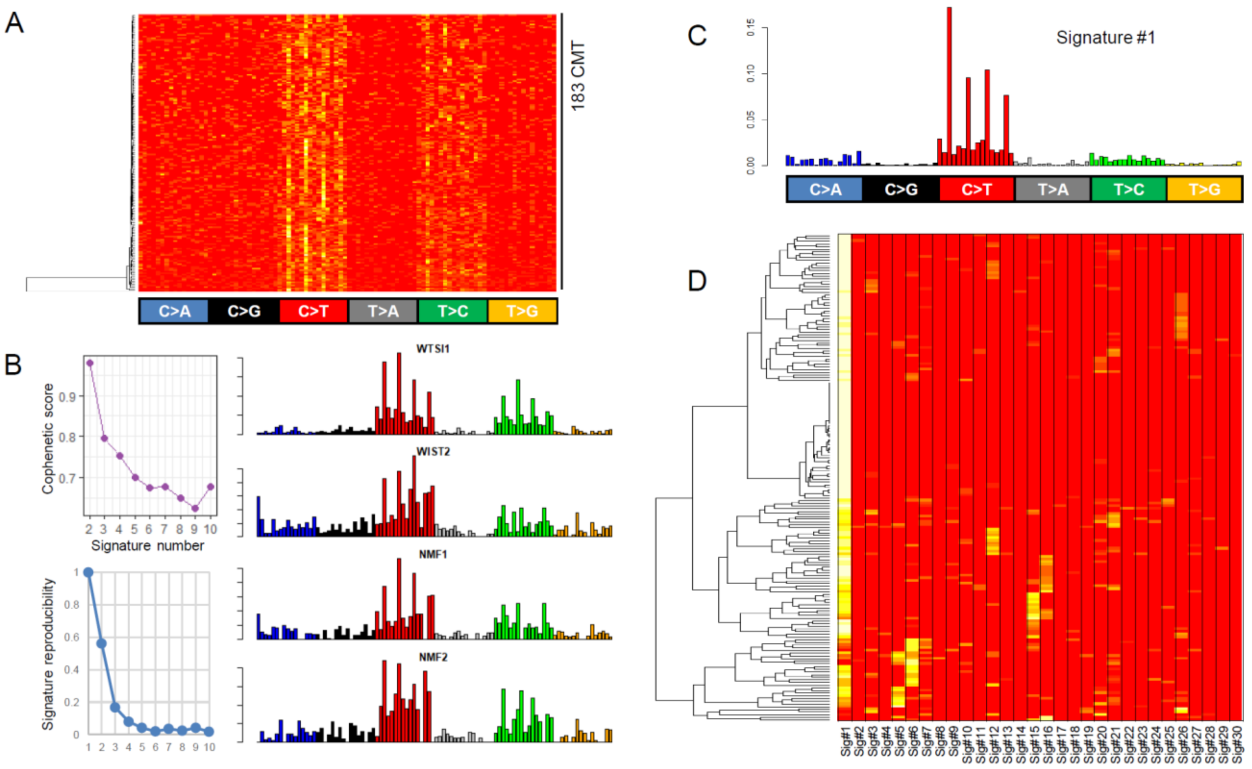

Supplementary Figure 4. Mutation signatures of CMT. (A) The frequencies of 96 trinucleotides of somatic mutations (*x*-axis) are shown for 183 CMTs (*y*-axis). C-to-T transitions are dominant mutations along with T-to-C transitions. (B) Two types of *de novo* mutation signatures are shown as driven by non-matrix factorization (NMF) and Wellcome Trust Sanger Institute (WTSI) mutation signature framework. (C) Signature #1 of COSMIC mutation signatures is shown for their relative frequencies in the 96 trinucleotide context. (D) The estimated levels of known 30 COSMIC mutation signatures are shown in a heatmap. The majority of the observed mutation signature is those from Signature #1.

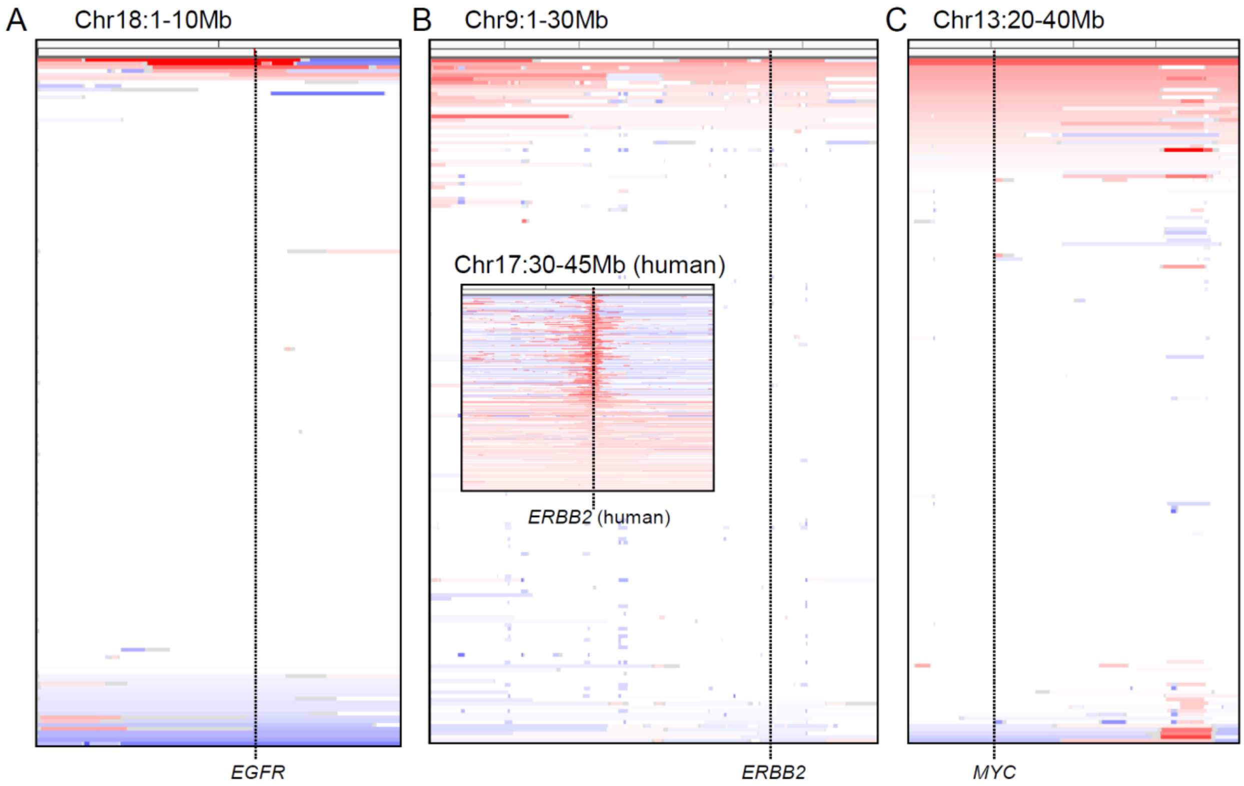

Supplementary Figure 5. Minimal amplification of *EGFR*, *ERBB2*, and *MYC*. (A) Copy number profiles at the *EGFR* locus (chr18:1 – 10Mb) is shown as a snapshot from the IGV browser. Red and blue represent chromosomal gains and losses, respectively. To visualize minimally amplified or deleted regions, the cases are sorted in order of their copy number ratios of the corresponding loci. (B) Similar snapshot for the *ERBB2* locus (chr9:1-30Mb). *ERBB2* shows a characteristic level of amplification, but is often separated from pter-gains, which is identified as GISTIC peaks. ERBB2 is not identified by GISTIC analysis. (C) The MYC locus also shows chromosomal gains for a number of cases, although a GISTIC peak is identified at a distal locus.

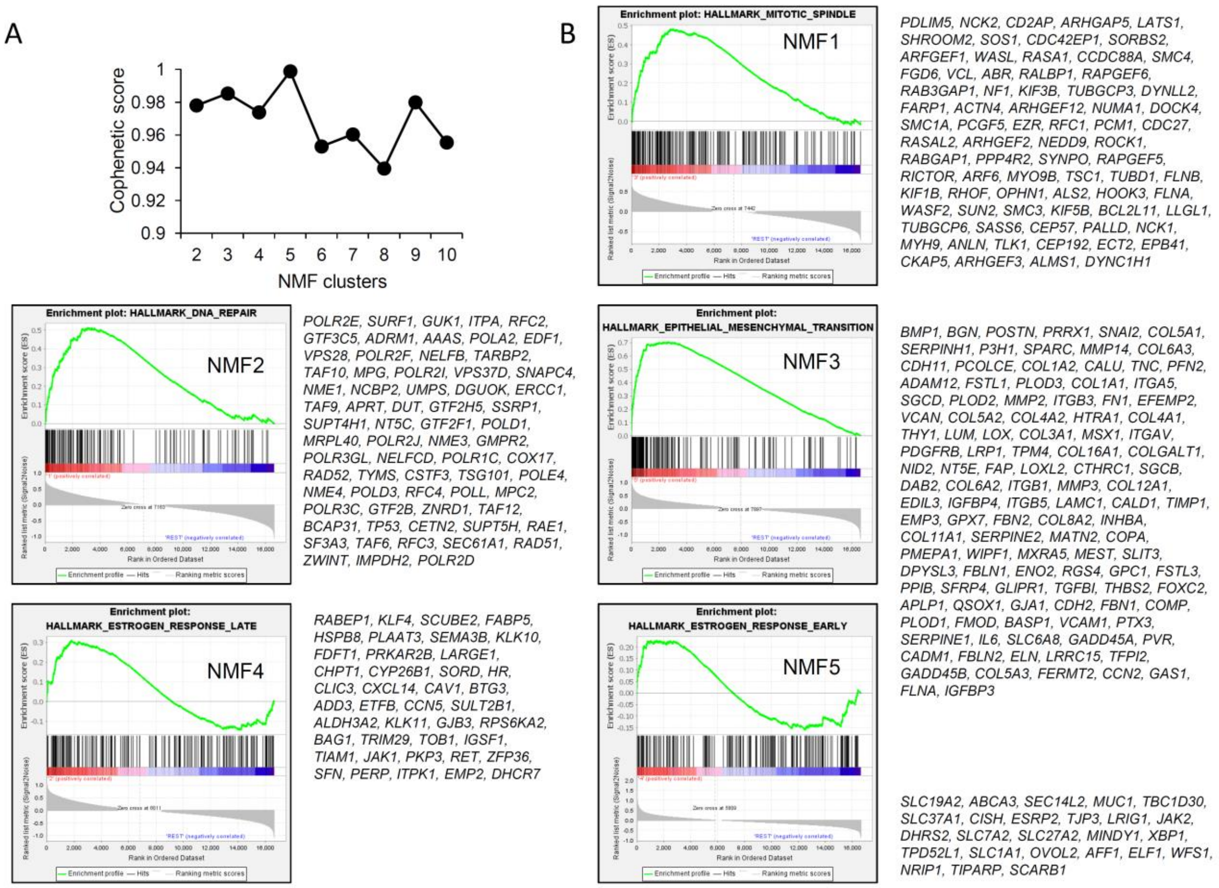

Supplementary Figure 6. Functional annotation of the five NMF clusters. (A) Cophenetic scores as stability measures are shown against the number of NMF clusters. (B) For the five NMF clusters, the enrichment plots of representative hallmark gene sets are shown with leading edge genes.

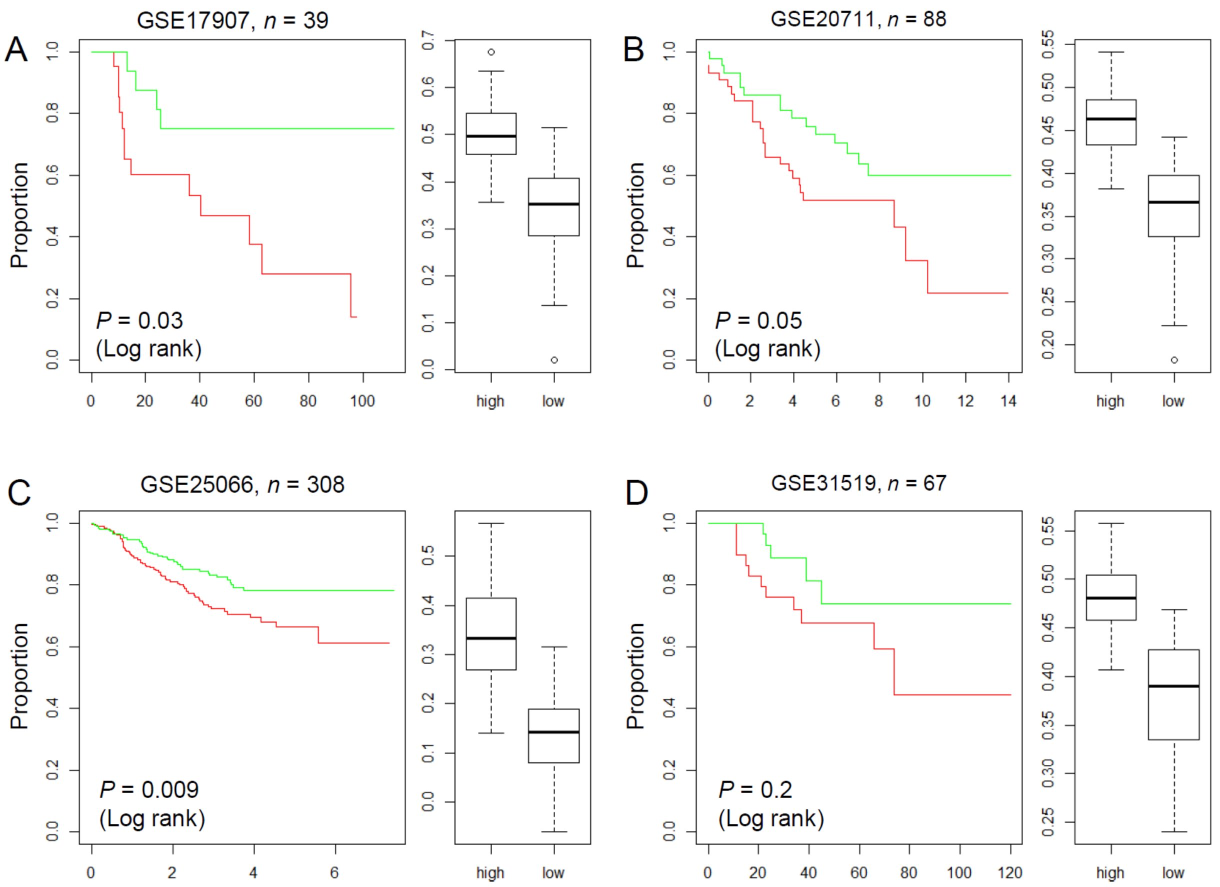

Supplementary Figure 7. NMF3 CMT subtypes in human breast cancers. (A) For 38 cases in GSE17907 as available in public GEO (Gene Expression Omnibus) database, Kaplan-Meier survival curves are for those with high and low NMF3 gene set scores (red and green, respectively). EMT scores are also shown. The significance was estimated by two-sided *U* tests**.** (B-D) Similarly shown for three additional cohorts.

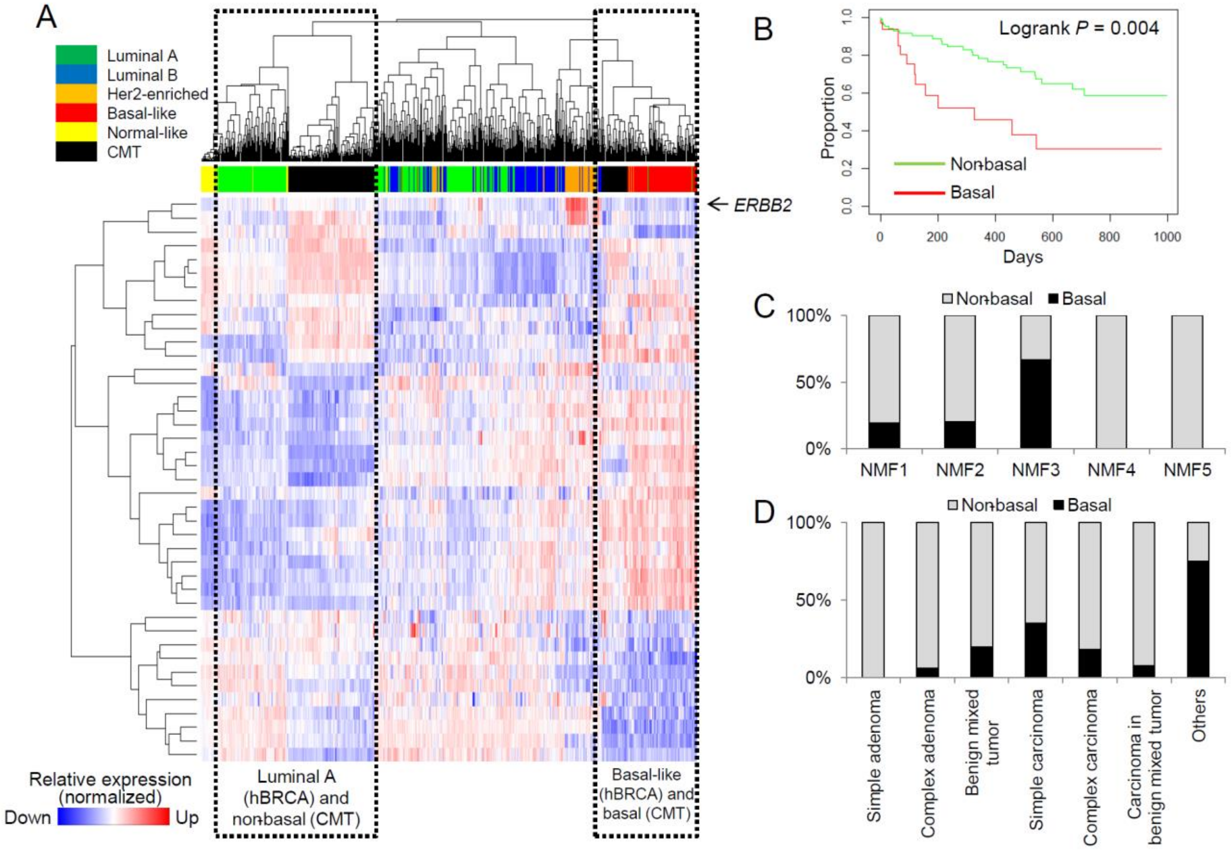

Supplementary Figure 8. Application of human breast cancer molecular taxonomy on the CMT cohort. (A) The merged gene expression profiles of human breast cancer and CMTs were subjected to hierarchical clustering revealing two major CMT subtypes. Non-basal CMTs and basal CMTs were co-segregated with luminal A and basal-like hBRCAs, respectively. (B) Kaplan-Meyer survival curves with log-rank tests show that basal CMTs have significantly shorter survival than non-basal CMTs (C and D). The NMF clusters and histology subtypes are shown against non-basal and basal CMTs, respectively.

Supplementary Tables

Supplementary Table 1. Genes with significant selection scores in CMT. dNdSCV scores corresponding to various types of nonsilent mutations (missense, nonsense, splicing mutations, and indels) are shown as output of dNdSCV algorithm. FDR (qglobal_cv) < 0.3 were significant. The numbers of substitutions of each class are those of synonymous (n_syn), missense (n_mis), nonsense (n_non), splicing (n_spl), and indel (n_ind). The maximum-likelihood estimates of the dN/dS ratios are also shown for missense (wmis), nonsense (wnon), splicing (wspl) and indels (wind). The *p* and *q* values are shown in each category and the global *q* values integrating all mutation types are shown as qglobal_cv.

gene_name	n_syn	n_mis	n_non	n_spl	n_ind	wmis_cv	wnon_cv	wspl_cv	wind_cv	pmis_cv	ptrunc_cv	pallsubs_cv	pind_cv	qmis_cv	qtrunc_cv	qallsubs_cv	pglobal_cv	qglobal_cv
PIK3CA	0	91	0	0	5	114.02	0.00	0.00	36.92	0.00	0.68	0.00	0.00	0.00	0.93	0.00	0.00	0.00
KRAS	0	16	0	0	3	116.51	0.00	0.00	125.28	0.00	0.87	0.00	0.00	0.00	0.93	0.00	0.00	0.00
TP53	0	12	2	1	3	37.16	107.63	107.63	61.66	0.00	0.00	0.00	0.00	0.00	0.13	0.00	0.00	0.00
PTEN	0	10	2	0	2	35.13	71.13	71.13	41.43	0.00	0.00	0.00	0.00	0.00	0.93	0.00	0.00	0.00
PIK3R1	0	2	0	0	10	3.42	0.00	0.00	108.71	0.15	0.72	0.33	0.00	0.68	0.93	0.94	0.00	0.00
BRK1	0	5	0	0	2	52.29	0.00	0.00	151.78	0.00	0.89	0.00	0.00	0.01	0.93	0.04	0.00	0.00
CALD1	0	0	0	0	8	0.00	0.00	0.00	86.49	0.27	0.76	0.52	0.00	0.68	0.93	0.94	0.00	0.00
TTN	29	12	1	0	3	0.19	0.23	0.23	0.67	0.00	0.06	0.00	0.46	0.00	0.93	0.00	0.00	0.02
SLC6A8	0	8	0	0	2	16.65	0.00	0.00	28.70	0.00	0.85	0.00	0.01	0.07	0.93	0.35	0.00	0.03
MPI	0	0	0	0	5	0.00	0.00	0.00	69.48	0.32	0.82	0.59	0.00	0.68	0.93	0.94	0.00	0.08
CREBBP	0	0	3	0	3	0.00	20.35	20.35	9.58	0.04	0.00	0.00	0.02	0.68	0.93	0.39	0.00	0.09
AKT1	0	8	0	0	1	17.01	0.00	0.00	16.41	0.00	0.78	0.00	0.06	0.07	0.93	0.32	0.00	0.13
CREBZF	1	1	0	0	4	2.36	0.00	0.00	82.43	0.48	0.87	0.77	0.00	0.68	0.93	0.94	0.00	0.16
DNAJC2	0	0	0	3	1	0.00	42.44	42.44	12.57	0.33	0.00	0.00	0.07	0.68	0.93	0.39	0.00	0.22

Supplementary Table 2. List of GISTIC Peaks.

GISTIC peaks	Descriptor	Chr	Start	End	Interval (bp)	q values	Cancer-related genes
**Amplification Peak 1**	6.6	chr6	39093701	40024299	930598	0.0002	AXIN1 (194kb), TSC2 (228kb), TRAF7 (307kb)
**Amplification Peak 2**	7.7	chr7	80947801	80974532	26731	0.0000	
**Amplification Peak 3**	8.8	chr8	71335601	74330416	2994815	0.0001	AKT1
**Amplification Peak 4**	9.9	chr9	1	2066299	2066298	0.0076	ASPSCR1, RNF213, CANT1 (375kb)
**Amplification Peak 5**	12.12	chr12	23834701	23888899	54198	0.0019	
**Amplification Peak 6**	13.13	chr13	36502701	37946999	1444298	0.0000	RECQL4, PHOX2B (921kb)
**Amplification Peak 7**	14.14	chr14	1	914699	914698	0.0000	
**Amplification Peak 8**	14.14	chr14	40075101	40493399	418298	0.0000	HOXA9, HOXA11, HOXA13, HNRNPA2B1 (573kb), JAZF1 (709kb)
**Amplification Peak 9**	18.18	chr18	1	6453799	6453798	0.0030	IKZF1, EGFR,
**Amplification Peak 10**	18.18	chr18	25494401	25878499	384098	0.0016	HRAS
**Amplification Peak 11**	18.18	chr18	45704401	46491299	786898	0.0000	CARS (516kb), EXT2 (720kb)
**Amplification Peak 12**	20.2	chr20	56416601	58134056	1717455	0.0016	TCF3, STK11, FSTL3, MAP2K2 (951kb)
**Amplification Peak 13**	24.24	chr24	46131401	47698779	1567378	0.0000	SS18L1
**Amplification Peak 14**	25.25	chr25	50284301	51628933	1344632	0.0024	
**Amplification Peak 15**	26.26	chr26	13415701	14523499	1107798	0.0000	
**Amplification Peak 16**	31.31	chr31	37738701	39514299	1775598	0.0001	U2AF1 (475kb)
**Amplification Peak 17**	34.34	chr34	10230801	11464099	1233298	0.0018	TERT, SDHA (501kb)
**Amplification Peak 18**	36.36	chr36	22153701	22331799	178098	0.0000	
**Deletion Peak 1**	1.1	chr1	1.16E+08	1.17E+08	763198	0.0001	
**Deletion Peak 2**	2.2	chr2	3743201	6074399	2331198	0.0000	
**Deletion Peak 3**	2.2	chr2	54238201	54767999	529798	0.0000	
**Deletion Peak 4**	3.3	chr3	24143401	26948299	2804898	0.0000	
**Deletion Peak 5**	3.3	chr3	89299901	91889043	2589142	0.0000	
**Deletion Peak 6**	4.4	chr4	19564101	19768699	204598	0.0000	TET1,
**Deletion Peak 7**	4.4	chr4	70507301	71586999	1079698	0.0000	
**Deletion Peak 8**	5.5	chr5	8664301	32756099	2.4E+07	0.0056	ARHGEF12,CBL,DDX6,KMT2A,PCSK7,PAFAH1B2,ZBTB16,POU2AF1,DDX10,ATM,BIRC3,RABEP1,TP53,
**Deletion Peak 9**	5.5	chr5	46530301	47661599	1131298	0.0000	
**Deletion Peak 10**	5.5	chr5	82425401	82642299	216898	0.0001	
**Deletion Peak 11**	6.6	chr6	49475701	50181599	705898	0.0016	
**Deletion Peak 12**	7.7	chr7	10620301	11087799	467498	0.0000	
**Deletion Peak 13**	7.7	chr7	80555201	80974532	419331	0.0000	
**Deletion Peak 14**	8.8	chr8	1	1711299	1711298	0.0000	TRIP11,
**Deletion Peak 15**	9.9	chr9	5076501	5425699	349198	0.0001	
**Deletion Peak 16**	9.9	chr9	12158901	12902299	743398	0.0000	DDX5,
**Deletion Peak 17**	11.11	chr11	22510301	22930099	419798	0.0000	
**Deletion Peak 18**	11.11	chr11	40668601	41568699	900098	0.0000	CDKN2A,
**Deletion Peak 19**	12.12	chr12	33036601	35662599	2625998	0.0019	
**Deletion Peak 20**	14.14	chr14	998801	1233699	234898	0.0000	
**Deletion Peak 21**	15.15	chr15	14032101	15544599	1512498	0.0000	MUTYH,
**Deletion Peak 22**	15.15	chr15	37280901	38133299	852398	0.0000	
**Deletion Peak 23**	16.16	chr16	9498901	9741799	242898	0.0020	KIAA1549,
**Deletion Peak 24**	16.16	chr16	13750301	14366799	616498	0.0007	
**Deletion Peak 25**	17.17	chr17	15998101	18498299	2500198	0.0000	
**Deletion Peak 26**	17.17	chr17	59687401	59942599	255198	0.0031	ARNT,
**Deletion Peak 27**	18.18	chr18	14811201	14854399	43198	0.0000	
**Deletion Peak 28**	18.18	chr18	41306101	42207299	901198	0.0000	
**Deletion Peak 29**	19.19	chr19	4980601	13198399	8217798	0.0000	
**Deletion Peak 30**	20.2	chr20	46914401	48090999	1176598	0.0000	BRD4,
**Deletion Peak 31**	21.21	chr21	31730801	33510099	1779298	0.0000	LMO1,
**Deletion Peak 32**	22.22	chr22	5648701	7138299	1489598	0.0000	
**Deletion Peak 33**	23.23	chr23	3806701	4595499	788798	0.0001	
**Deletion Peak 34**	24.24	chr24	25559601	25775399	215798	0.0000	
**Deletion Peak 35**	25.25	chr25	1	1450699	1450698	0.0000	FOXO1,
**Deletion Peak 36**	25.25	chr25	34315001	35034799	719798	0.0000	
**Deletion Peak 37**	26.26	chr26	6528201	7020099	491898	0.0002	ZCCHC8,CLIP1,
**Deletion Peak 38**	26.26	chr26	24945201	27052199	2106998	0.0000	
**Deletion Peak 39**	26.26	chr26	37291901	38964690	1672789	0.0000	PTEN,FAS,
**Deletion Peak 40**	27.27	chr27	5637001	6148599	511598	0.0099	
**Deletion Peak 41**	28.28	chr28	8468701	8844599	375898	0.0001	
**Deletion Peak 42**	29.29	chr29	16110801	16620499	509698	0.0000	
**Deletion Peak 43**	30.3	chr30	7640901	7907899	266998	0.0006	
**Deletion Peak 44**	31.31	chr31	36736601	39895921	3159320	0.0002	U2AF1,
**Deletion Peak 45**	33.33	chr33	29084401	31377067	2292666	0.0000	TFRC,
**Deletion Peak 46**	35.35	chr35	1	26524999	2.7E+07	0.0085	IRF4,DEK,
**Deletion Peak 47**	35.35	chr35	25967801	26524999	557198	0.0085	
**Deletion Peak 48**	36.36	chr36	1	30810995	3.1E+07	0.0000	ACVR1,CHN1,HOXD11,NFE2L2,
**Deletion Peak 49**	37.37	chr37	11056401	11895899	839498	0.0018	

Supplementary Table 3. GSEA results of five NMF metagene signatures

	Gene set (Hallmark, MSigDB)	SIZE	ES	NES	NOM p-val	FDR q-val	FWER p-val
**NMF1**	HALLMARK_MITOTIC_SPINDLE	190	0.48	1.61	0.027	0.66	0.416
	HALLMARK_TGF_BETA_SIGNALING	44	0.54	1.56	0.049	0.434	0.487
**NMF2**	HALLMARK_DNA_REPAIR	130	0.51	1.7	0.015	0.247	0.245
**NMF3**	HALLMARK_EPITHELIAL_MESENCHYMAL_TRANSITION	183	0.7	2.24	0	0	0
	HALLMARK_ANGIOGENESIS	32	0.72	2.18	0	0.001	0.001
	HALLMARK_APICAL_JUNCTION	180	0.37	1.55	0.017	0.317	0.46
	HALLMARK_GLYCOLYSIS	189	0.38	1.55	0.042	0.384	0.45
**NMF4**	HALLMARK_ESTROGEN_RESPONSE_LATE	182	0.31	1.43	0.014	0.586	0.677
	HALLMARK_HEME_METABOLISM	174	0.36	1.5	0.024	0.544	0.566
	HALLMARK_FATTY_ACID_METABOLISM	141	0.55	1.72	0.034	0.387	0.207
	HALLMARK_ADIPOGENESIS	179	0.54	1.69	0.041	0.243	0.244
**NMF5**	HALLMARK_ESTROGEN_RESPONSE_EARLY	184	0.23	1	0.433	1	0.978
