# Supplementary material for: Cross-species oncogenic signatures of breast cancer in canine mammary tumors
Source: Nat Commun. 2020 Jul 17;11:3616. doi: 10.1038/s41467-020-17458-0 (PMC7367841; doi:10.1038/s41467-020-17458-0)
Supplement: Supplementary file 3 — Description of Additional Supplementary Files [file 41467_2020_17458_MOESM3_ESM.pdf]

Description of Additional Supplementary Files

File Name: Supplementary Data 1
Description: Clinical information of the cohort. A total of 191 CMT in the study cohort are shown with available clinical information.

File Name: Supplementary Data 2
Description: Sequencing information of WES. The number of total reads sequenced and the mapping rates are shown.

File Name: Supplementary Data 3
Description: Sequencing information of RNA-seq.

File Name: Supplementary Data 4
Description: Exonic mutations of 183 CMT. A total of 10,855 exonic SNVs and indels are listed with genomic coordinates based on CanFam3.1.

File Name: Supplementary Data 5
Description: Germline variants of CMT genomes.

File Name: Supplementary Data 6
Description: Mutation frequencies of genes in CMT and human breast cancers. The frequency (%) of non-silent mutations in benign-mailgnant CMTs are shown with those of TCGA breast cancers.
