# Supplementary material for: Cross-species oncogenic signatures of breast cancer in canine mammary tumors
Source: Nat Commun. 2020 Jul 17;11:3616. doi: 10.1038/s41467-020-17458-0 (PMC7367841; doi:10.1038/s41467-020-17458-0)
Supplement: Supplementary file 10 — Reporting Summary [file 41467_2020_17458_MOESM10_ESM.pdf]

nature research

Corresponding author(s): Sangwoo Kim

Last updated by author(s): Jun 22, 2020

Reporting Summary

Nature Research wishes to improve the reproducibility of the work that we publish. This form provides structure for consistency and transparency in reporting. For further information on Nature Research policies, see Authors & Referees and the Editorial Policy Checklist .

Statistics

For all statistical analyses, confirm that the following items are present in the figure legend, table legend, main text, or Methods section.

n/a	Confirmed	
☐	☒	The exact sample size (*n*) for each experimental group/condition, given as a discrete number and unit of measurement
☐	☒	A statement on whether measurements were taken from distinct samples or whether the same sample was measured repeatedly
☐	☒	The statistical test(s) used AND whether they are one- or two-sided<br>*Only common tests should be described solely by name; describe more complex techniques in the Methods section.*
☒	☐	A description of all covariates tested
☐	☒	A description of any assumptions or corrections, such as tests of normality and adjustment for multiple comparisons
☐	☒	A full description of the statistical parameters including central tendency (e.g. means) or other basic estimates (e.g. regression coefficient) AND variation (e.g. standard deviation) or associated estimates of uncertainty (e.g. confidence intervals)
☐	☒	For null hypothesis testing, the test statistic (e.g. *F*, *t*, *r*) with confidence intervals, effect sizes, degrees of freedom and *P* value noted<br>*Give P values as exact values whenever suitable.*
☒	☐	For Bayesian analysis, information on the choice of priors and Markov chain Monte Carlo settings
☒	☐	For hierarchical and complex designs, identification of the appropriate level for tests and full reporting of outcomes
☐	☒	Estimates of effect sizes (e.g. Cohen's *d*, Pearson's *r*), indicating how they were calculated

Our web collection on statistics for biologists contains articles on many of the points above.

Software and code

Policy information about availability of computer code

Data collection	No special software was used
Data analysis	BWA-MEM2, Picard v2.2, Samtools v1.9, GATK v4.0.10.1, TopHat v2.0.9, CuffLinks v2.1.1, Varscan v2.4.3, GISTIC2 v.2.0.22, CBS/R package v.1.62, NMF/R package v.0.22, GSEA v.4.0, CIBERSORT v.1.04, SnpEFF v.4.3t, ClinVar build 2020-03-10, GATK-HC v4.1.5.0, Crossmap v.0.2.9,

For manuscripts utilizing custom algorithms or software that are central to the research but not yet described in published literature, software must be made available to editors/reviewers. We strongly encourage code deposition in a community repository (e.g. GitHub). See the Nature Research guidelines for submitting code & software for further information.

Data

Policy information about availability of data

All manuscripts must include a data availability statement. This statement should provide the following information, where applicable:

- Accession codes, unique identifiers, or web links for publicly available datasets
- A list of figures that have associated raw data
- A description of any restrictions on data availability

Raw DNA and RNA sequencing data are publicly available in Sequence Read Archive (SRA) with the accession numbers 159481 (DNA-seq) and 159466 (RNA-seq). Gene expression values are available in Gene Expression Omnibus (GEO) with the accession number GSE119810. The lists of germ line and somatic mutations are available in Supplementary Tables. Single nucleotide variants and their allele frequencies are available at the Dog Genome SNP Database (DogSD) from the iDOG website (http://bigd.big.ac.cn/idog/).

Field-specific reporting

Please select the one below that is the best fit for your research. If you are not sure, read the appropriate sections before making your selection.

- [x] Life sciences
- [ ] Behavioural & social sciences
- [ ] Ecological, evolutionary & environmental sciences

For a reference copy of the document with all sections, see nature.com/documents/nr-reporting-summary-flat.pdf

Life sciences study design

All studies must disclose on these points even when the disclosure is negative.

Sample size	The sample size has been chosen to discovery recurrent somatic mutation with >5% allele frequency.
Data exclusions	There is no data that were excluded from analyses.
Replication	There is no replication in the study. The is a discovery study that reports the identified genetic mutations.
Randomization	The experiments were not randomized in our pilot study. Based on our design, all the samples were grouped into benign and malignant tumors according to their biological and histological features.
Blinding	The investigators were not blinded to allocation during experiments. This is a discovery study that reports the identified genetic features.

Reporting for specific materials, systems and methods

We require information from authors about some types of materials, experimental systems and methods used in many studies. Here, indicate whether each material, system or method listed is relevant to your study. If you are not sure if a list item applies to your research, read the appropriate section before selecting a response.

Materials & experimental systems

n/a	Involved in the study	
x		Antibodies
x		Eukaryotic cell lines
x		Palaeontology
	x	Animals and other organisms
	x	Human research participants
x		Clinical data

Methods

n/a	Involved in the study	
x		ChIP-seq
x		Flow cytometry
x		MRI-based neuroimaging

Animals and other organisms

Policy information about studies involving animals; ARRIVE guidelines recommended for reporting animal research

Laboratory animals	This study did not involve laboratory animals
Wild animals	This study did not involve wild animals.
Field-collected samples	Environment parameters are now known and/or not relevant to this study. The involve animals are pet-dogs, which are privately-owned by donors. The tumor samples have been stored in FFPE condition after removed from the dogs.
Ethics oversight	Tissue samples were obtained as a part of routine diagnostic procedures, and blood samples were collected for research following the guidelines of and approval from the Institutional Animal Care and Use Committee of Konkuk University (KU16106 and KU17162).

Note that full information on the approval of the study protocol must also be provided in the manuscript.

Human research participants

Policy information about studies involving human research participants

Population characteristics	We used publicly available gene expression profiles (GSE17907, GSE20711, GSE25066 and GSE31519).<br>No new recruitment was done for this study. The population characteristics are described in the original studies
Recruitment	Recruitment has been done in the corresponding original studies. No recruitment of selection has been done in this study.

Ethics oversight	Ethical review and approval was done in the corresponding original studies. We only used the publicly available gene expression values.

Note that full information on the approval of the study protocol must also be provided in the manuscript.
